# Protective effects of *Arctium lappa L*. root extracts (AREs) on high fat diet induced quail atherosclerosis

**DOI:** 10.1186/s12906-016-0987-2

**Published:** 2016-01-08

**Authors:** Zhi Wang, Ping Li, Chenjing Wang, Qixiao Jiang, Lei Zhang, Yu Cao, Weizhen Zhong, Chunbo Wang

**Affiliations:** 1 The Affiliated Hospital of Qingdao University, 16 Jiangsu Road, Qingdao, 266003 Shandong China; 2 Department of Pharmacology, Qingdao University Medical College, Boya Building Room 422, 308 Ningxia Road, Qingdao, 266071 Shandong China

**Keywords:** *Arctium lappa L*. root extracts, Atherosclerosis, High fat diet, Hypolipidemic effect, Anti-oxidant effect

## Abstract

**Background:**

This study was designed to evaluate the protective effects of Arctium lappa L. root extracts (AREs) from different extraction methods (aqueous, ethanol, chloroform and flavone) on atherosclerosis.

**Methods:**

Quails (Coturnix coturnix) were subjected to high fat diet, with or without one of the four different AREs or positive control simvastatin. Blood samples were collected before treatment, after 4.5 weeks or ten weeks to assess lipid profile (Levels of total cholesterol (TC), Triacylglycerol (TG), low-density lipoprotein (LDL) and high-density lipoprotein (HDL)). After ten weeks, the serum levels of nitric oxide (NO) as well as antioxidant and pro-oxidative status (Levels of malondialdehyde (MDA), superoxide dismutase (SOD), catalase (CAT), glutathione (GSH), nicotinamide adenine dinucleotide phosphate (NADPH) and glutathione peroxidase (GSH-Px)) were measured. Furthermore, aortas were collected after ten weeks treatment, aorta lipid contents (TC, TG and LDL) were assessed, and histology was used to confirm atherosclerotic changes.

**Results:**

The results indicated that high fat diet significantly deteriorated lipid profile and antioxidant status in quail serum, while all the extracts significantly reverted the changes similar to simvastatin. Aorta lipid profile assessment revealed similar results. Histology on aortas from quails treated for ten weeks confirmed atherosclerotic changes in high fat diet group, while the extracts significantly alleviated the atherosclerotic changes similar to simvastatin. Among the different extracts, flavones fraction exerted best protective effects.

**Conclusions:**

Our data suggest that the protective effects of AREs were medicated via hypolipidemic and anti-oxidant effects. Underlying molecular mechanisms are under investigation.

## Background

Cardiovascular diseases are the leading causes of morbidity and mortality among adult population throughout the world [1, 2]. Atherosclerosis is the primary cause of cardiovascular disease, leading to the occlusion of the arteries, resulting in ischemia in vital organs such as heart and brain and subsequent death. The pathogenesis of atherosclerosis is a complex and chronic multifactorial process and remains a popular topic of research. Identified risk factors are mainly metabolic disorders such as dyslipidemia, obesity and diabetes, in which inflammation and oxidative stress seems to be the common routes to atherogenesis [3]. The initial step of oxidative-stress induced damage to endothelium was extensively studied, which involves macrophage and oxidized low-density lipoprotein (ox-LDL) [4, 5]. Atherosclerosis have well-characterized pathological changes include dysfunction of vascular endothelium, differentiation of monocyte into macrophages, conversion of macrophages into foam cells and proliferation of smooth muscle cell [4, 5].

The investigation of contributing factors of atherosclerosis is an ongoing hot research topic. Hyperlipidemia, especially hypercholesterolemia was found to play an important role in the initiation and progression of atherosclerosis [2]. Among the negative impacts of hyperlipidemias, it has been well characterized that hyperlipidemia could induce overproduction of reactive oxygen species in endothelial cells, smooth muscle cells and macrophages, subsequently resulting in oxidative stress and lipid peroxidation in both humans and animals [6, 7]. Thus, oxidative stress has been implicated in the pathogenesis of atherosclerosis, as an independent stage of the disease progression [8].

*Arctium lappa L*, commonly known as burdock or bardana, is a perennial plant of the Asteraceae (Compositae) family. It has been reported that the extract and fractions of *Arctium lappa L* roots are used for its colitis prophylactic [9], gastro-protective [10], anti-sterility [11], antimicrobial [12] and anti-proliferative effects [13, 14]. A recent study reported the anti-inflammatory and free radical scavenging activities of *Arctium lappa* root extracts (AREs) [15]. The anti-oxidant capability [16] suggested the protective potential of AREs against atherosclerosis. The present study utilized high fat diet induced atherosclerosis model in quail and focused on the protective effects of AAE on high fat diet induced atherosclerosis. The high fat diet quail model ad been used in multiple studies [17–19], features diet induced atherosclerosis and does not require genetic modifications, which is a good mimic of real-life atherosclerosis.

## Methods

### Materials

*Arctium lappa L*. Root was purchased from Anqiu vegetable farm center (Weifang, Shandong, China) and identified by Dr. Jingying Sun (Shandong Academy of Medicine Pharmacy Institute). *Arctium lappa L*. is a well established traditional Chinese medicine herb, since the material used in this study met the quality standards set by the Shandong Food and Drug Administration (SDFDA), no further specimen deposit was performed. Simvastatin was purchased from Hainan Pharmaceutical Factory Co., Ltd. (Hainan, China) (20 mg/Tablet, Batch number: 130406). 1,4-dioxane was purchased from Tianjin Ruijin Chemicals Co. Ltd. (Tianjin, China). Isopropyl alcohol (ISO) was obtained from Tianjin Fuyu Fine Chemical Co., LTD. (Tianjin, China). Nitric oxide (NO), malondialdehyde (MDA), superoxide dismutase (SOD), catalase (CAT), glutathione (GSH), nicotinamide adenine dinucleotide phosphate (NADPH) and glutathione peroxidase (GSH-Px) assay kits were purchased from Jiancheng Institute of Biological Engineering (Nanjing, China).

### Preparation of AREs

#### Preparation of the ethanol extract (AEE)

The dried roots of A. Lappa (0.6 kg) were soaked in 4 L of 80 % ethanol for 24 h, and then reflux extracted twice with 80 and 60 % ethanol for 5 h respectively. The resulting residue was dried and used for next extraction. The organic solutions were combined and concentrated under reduced pressure to give 301.8 g of the ethanol extract. 231.6 g starch was added to facilitate the formation of powder, resulting in a total of 533.4 g AEE.

#### Preparation of the aqueous extract (AAE)

The dried pretreated sample was dipped in distilled water (9 L) and boiled twice for 2 h. The combined filtrate was then concentrated under reduced pressure and evaporated to dryness and the yield of the aqueous extract is 59.1 g. 21.5 g starch was added, resulting in a total of 80.6 g AAE.

#### Preparation of the chloroform fraction (ACE)

The dried roots of A. Lappa (10 kg) were reflux extracted twice with methanol for 5 h. The methanol solution was contration under reduced pressure to obtain a residue. The extract was suspended in water (6 L) and extracted with chloroform (6 L × 3 times). The resulting fraction was collected and then concentrated in vacuo to afford 48.3 g the chloroform fraction. 68.6 g starch was added to form powder. 116.9 g ACE was the final total yield.

#### Preparation of the flavones extract (AFE)

The remaining solution from chloroform extract was heated to evaporate the organic solvent. Cooled solution was added into column loading-treated Macroporous resin DM-130 for absorption, and then washed with water and 10 % ethanol, respectively, to get rid of impurities. The total flavonoids in column was eluted with 80 % ethanol and dried in a vacuum condition until powder was formed (157.7 g). 17.1 g starch was added to form a total of 174.8 g AFE.

### Identification of the AREs

Extracted AREs were analyzed in a previous study carried out by our group. Refer to this work for information about detailed compositions and identified specific chemicals in different AREs [20].

### Animal treatment and sample collection

Male quails (3 weeks old, body weight about 100 g) were purchased from Lanke Poultry Breeding Center (Jimo, Qingdao, China). All experimental protocols were approved by the Institutional Animal Use and Care Committee of Qingdao University (Qingdao, China). Quails were kept on a 12 h day/night lighting schedule and had access to standard quail chow and water *ad libitum*. After one week environment adaption, the quails were randomly divided into treatment groups. Due to limited handling capacity, two separated batches were used. First batch included: control group, model group, positive control group and AAE groups (AAE 0.75, 1.5 or 3 g/kg/day); second batch included: control group, model group, positive control group, AEE groups (AEE 1 or 2 g/kg/day), ACE groups (ACE 100 or 200 mg/kg/day) and AFE groups (150 or 300 mg/kg/day). Quails in the control group were fed standard chow (0 % cholesterol). Quails in all other groups were fed with high fat diet (1 % cholesterol and 14 % pork oil, w/w), along with specified treatments via gavage. After 4.5 weeks treatment, 2 mL blood were collected from quail right jugular vein. After 10 weeks treatment, terminal body weights were recorded, then venous blood sample (3 mL) was taken from the right jugular vein of each quail. Collected blood samples were incubated at 37 °C for 10 min and then centrifuged at 3000 rpm for 10 min, resulting serum samples were collected and archived at -80 °C until further analysis. The animals were then sacrificed and aorta were dissected for further analysis.

### Serum lipid profile assessment

Serum collected at the beginning of the experiment, after 4.5 weeks treatment and after ten weeks treatment were subjected to automatic biochemistry analyzer Beckman AU5400 (Brea, CA, US) for serum lipid profile assessment. The levels of serum total cholesterol (TC), low density lipoprotein (LDL), and high density lipoprotein (HDL) were assessed.

### Anti-oxidant and pro-oxidative status assessment in serum

All the biochemical parameters (NO, MDA, SOD, GSH, NADPH and GSH-Px) were measured with commercial kits (Nanjing Jiancheng Bioengineering Institute, Jiangsu, China) following manufacturer’s protocols. All the kits were based on colorimetric methods, and were carried out on a spectrophotometer 722E (Shanghai Spectrum Instruments, Shanhai, China).

### Histology of quail aorta

Dissected aortas (aorta arch) were fixed in 4 % formaldehyde for 24 h, embedded in paraffin, and then sectioned at 6 μm on a rotary microtome (Leica RM2016, Wetzlar, Germany). Hematoxylin and eosin (Beyotime, Jiangsu, China) staining was performed following manufacturer’s protocol. Pictures were taken with a microscope (Olympus BX51, Tokyo, Japan) and analyzed with ImageJ (NIH, US). The ratio of atherosclerotic area to total aorta area was calculated as an assessment of atherosclerosis.

### Transmission electronic microscopy on quail aorta

Dissected aortas were fixed in 5 % glutaraldehyde for 24 h, dehydrated with graded ethanol, embedded with epoxy resin 618, sectioned and observed under a transmission electronic microscope JEM-1200EX (JEOL, Tokyo, Japan).

### Aorta lipid profile assessment

Aortas were homogenized in 1 % 1,4-dioxane. Samples were then incubated in a 37 ^o^C incubator shaker for 72 h. For each sample, 1 mL of supernatant was collected and dried, and then dissolved in 50 μL of isopropanol. Lipid profile was then assessed by subjecting dissolved samples to automatic biochemical analyzer Beckman AU5400 (Brea, CA, US).

### Statistical analysis

Drawing lots method was used to ensure that quails were randomly assigned into each treatment groups. SPSS 17.0 was used to perform statistical analysis. All data were expressed as mean ± standard derivation. Normal distribution was confirmed with Levene’s test, then one-way analysis of variance (ANOVA) was performed. When *P*-values were less than 0.05, the statistical significance was determined and post-hoc least significant difference (LSD) tests were performed for the differences among groups.

## Results

### Effect of AREs on serum lipids

To assess the lipid-lowering effect of AREs, serum TG, TC, LDL and HDL were measured at set time point. After 4.5 weeks of treatment, when compared with control group, levels of TC, TG and LDL in the high fat diet group increased significantly, while level of HDL decreased significantly. Positive control drug, simvastatin, effectively reverted all the changes. Low dose (0.75 g/kg/day) AAE significantly decreased TG level comparing to high fat diet group, while no significant effect on the levels of TC, LDL or HDL were found at this dose. However, higher doses (1.5 and 3 g/kg/day) both significantly decreased TC, TG and LDL levels and increased HDL levels relative to high fat diet group (Tables 5, 6, 7 and 8). After 10 weeks of treatment, the changes in levels of TC, TG, LDL and HDL further increased in the high fat diet group, while both simvastatin and AAE at all doses significantly reverted the changes. At this time point, AAE exhibited a nice dose-dependent manner for TC and LDL (Tables 1, 2, 3 and 4).

**Table 1:** Serum lipid profile of high fat diet-fed quails treated with AAE after four and a half weeks (mM)

Treatment (/kg/day)	Total cholesterol	Triacylglycerol	LDL	HDL
Control	5.64 ± 0.79	1.15 ± 0.24	1.41 ± 0.21	3.56 ± 0.46
Model	7.09 ± 0.68^a^	1.55 ± 0.33^a^	1.80 ± 0.27^a^	2.76 ± 0.35^a^
Simvastatin 15 mg	5.95 ± 0.63^b^	1.19 ± 0.22^b^	1.45 ± 0.11^b^	3.14 ± 0.36^b^
AAE 0.75 g	6.60 ± 0.41^c^	1.16 ± 0.31^b^	1.70 ± 0.17^c^	3.11 ± 0.29
AAE 1.5 g	6.20 ± 0.46^bc^	1.09 ± 0.25^b^	1.58 ± 0.26^b^	3.18 ± 0.45^b^
AAE 3 g	5.34 ± 0.37^bcde^	1.06 ± 0.19^b^	1.46 ± 0.19^bd^	3.25 ± 0.48^b^

^a^statistically different from control group (*P* < 0.05)

^b^statistically different from model group (*P* < 0.05)

^c^statistically different from simvastatin group (*P* < 0.05)

^d^statistically different from AAE 0.75 g group (*P* < 0.05)

^e^statistically different from AAE 1.5 g group (*P* < 0.05)

Male quails(three weeks old at the beginning of experiments, four weeks old at the beginning of treatments) were subjected to high fat diet (1 % cholesterol and 14 % pork oil, w/w) for a total of ten weeks. Venous blood were collected from right jugular veins after four and a half weeks and ten weeks treatment. Serums were subjected to automatic biochemistry analyzer Beckman AU5400 (Brea, CA, US) for total cholesterol, triacylglycerol, low density lipoprotein (LDL) and high density lipoprotein (HDL). Data are shown as mean ± standard derivation, *N* = 10

**Table 2:** Serum lipid profile of high fat diet-fed quails treated with AAE after ten weeks (mM)

Treatment (g/kg/day)	Total cholesterol	Triacylglycerol	LDL	HDL
Control	5.65 ± 0.70	1.10 ± 0.17	1.41 ± 0.20	3.86 ± 0.55
Model	7.79 ± 0.98^a^	1.79 ± 0.28^a^	2.03 ± 0.27^a^	2.47 ± 0.36^a^
Simvastatin 15 mg	5.75 ± 0.40^b^	1.13 ± 0.25^b^	1.49 ± 0.10^b^	3.37 ± 0.43^b^
AAE 0.75 g	6.89 ± 0.36^bc^	1.48 ± 018^bc^	1.86 ± 0.12^bc^	3.00 ± 0.28^b^
AAE 1.5 g	6.10 ± 0.38^bcd^	1.28 ± 0.28^b^	1.62 ± 0.23^bd^	3.45 ± 0.41^bd^
AAE 3 g	5.14 ± 0.30^bcde^	1.03 ± 0.20^bcde^	1.43 ± 0.14^bde^	3.45 ± 0.52^bd^

^a^statistically different from control group (*P* < 0.05)

^b^statistically different from model group (*P* < 0.05)

^c^statistically different from simvastatin group (*P* < 0.05)

^d^statistically different from AAE 0.75 g group (*P* < 0.05)

^e^statistically different from AAE 1.5 g group (*P* < 0.05)

Male quails(three weeks old at the beginning of experiments, four weeks old at the beginning of treatments) were subjected to high fat diet (1 % cholesterol and 14 % pork oil, w/w) for a total of ten weeks. Venous blood were collected from right jugular veins after four and a half weeks and ten weeks treatment. Serums were subjected to automatic biochemistry analyzer Beckman AU5400 (Brea, CA, US) for total cholesterol, triacylglycerol, low density lipoprotein (LDL) and high density lipoprotein (HDL). Data are shown as mean ± standard derivation, *N* = 10

**Table 3:** Serum lipid profile of high fat diet-fed quails treated with AEE, ACE or AFE after four and a half weeks (mM)

Treatment (/kg/day)	Total cholesterol	Triacylglycerol	LDL	HDL
Control	5.60 ± 0.32	1.07 ± 0.23	1.30 ± 0.35	3.71 ± 0.42
Model	6.69 ± 0.44^a^	1.74 ± 0.27 ^a^	1.95 ± 0.22^a^	2.30 ± 0.32^a^
Simvastatin 15 mg	5.72 ± 0.36^b^	1.37 ± 0.18 ^b^	1.37 ± 0.38^b^	3.61 ± 0.44^b^
AEE 1 g	6.57 ± 0.51^c^	1.53 ± 0.35	1.88 ± 0.24	2.53 ± 0.90^c^
AEE 2 g	6.58 ± 0.45^c^	1.85 ± 0.51^c^	1.84 ± 0.39	2.84 ± 0.43^bc^
ACE 100 mg	6.32 ± 0.75^c^	1.59 ± 0.46	1.89 ± 0.15	2.75 ± 0.38^c^
ACE 200 mg	6.71 ± 0.50^c^	1.66 ± 0.23	1.81 ± 0.28	2.69 ± 0.38^c^
AFE 150 mg	6.62 ± 0.52^c^	1.63 ± 0.44	1.88 ± 0.39	2.80 ± 0.66^bc^
AFE 300 mg	6.51 ± 0.49^c^	1.60 ± 0.50	1.91 ± 0.48	2.87 ± 0.46^bc^

^a^statistically different from control group (*P* < 0.05)

^b^statistically different from model group (*P* < 0.05)

^c^statistically different from simvastatin group (*P* < 0.05)

Male quails(three weeks old at the beginning of experiments, four weeks old at the beginning of treatments) were subjected to high fat diet (1 % cholesterol and 14 % pork oil, w/w) for a total of ten weeks. Venous blood were collected from right jugular veins after four and a half weeks and ten weeks treatment. Serums were subjected to automatic biochemistry analyzer Beckman AU5400 (Brea, CA, US) for total cholesterol, triacylglycerol, low density lipoprotein (LDL) and high density lipoprotein (HDL). Data are shown as mean ± standard derivation, *N* = 10

**Table 4:** Serum lipid profile of high fat diet-fed quails treated with AEE, ACE or AFE after ten weeks (mM)

Treatment (/kg/day)	Total cholesterol	Triacylglycerol	LDL	HDL
Control	5.51 ± 0.59	1.11 ± 0.13	1.15 ± 0.22	4.10 ± 0.47
Model	7.42 ± 0.57^a^	1.84 ± 0.37 ^a^	1.88 ± 0.22^a^	2.42 ± 0.34^a^
Simvastatin 15 mg	5.69 ± 0.34^b^	1.14 ± 0.22 ^b^	1.37 ± 0.07^b^	4.16 ± 0.75^b^
AEE 1 g	6.34 ± 0.79^c^	1.67 ± 0.30^c^	1.99 ± 0.32^c^	3.28 ± 0.62^bc^
AEE 2 g	5.96 ± 0.56^b^	1.21 ± 0.17 ^bd^	1.37 ± 0.22^bd^	3.62 ± 0.47^bc^
ACE 100 mg	7.20 ± 0.71^c^	1.72 ± 0.38^c^	1.80 ± 0.52^c^	2.60 ± 0.46^c^
ACE 200 mg	6.90 ± 0.80^c^	1.48 ± 0.45 ^c^	1.67 ± 0.36^c^	2.63 ± 0.38^c^
AFE 150 mg	6.04 ± 0.70^b^	1.33 ± 0.31 ^b^	1.51 ± 0.21^b^	3.11 ± 0.96^bc^
AFE 300 mg	5.87 ± 0.50^b^	1.06 ± 0.18 ^be^	1.31 ± 0.19^b^	3.78 ± 0.41^bce^

^a^statistically different from control group (*P* < 0.05)

^b^statistically different from model group (*P* < 0.05)

^c^statistically different from simvastatin group (*P* < 0.05)

^d^statistically different from AAE 1 g group (*P* < 0.05)

^e^statistically different from AFE 300 mg group (*P* < 0.05)

Male quails(three weeks old at the beginning of experiments, four weeks old at the beginning of treatments) were subjected to high fat diet (1 % cholesterol and 14 % pork oil, w/w) for a total of ten weeks. Venous blood were collected from right jugular veins after four and a half weeks and ten weeks treatment. Serums were subjected to automatic biochemistry analyzer Beckman AU5400 (Brea, CA, US) for total cholesterol, triacylglycerol, low density lipoprotein (LDL) and high density lipoprotein (HDL). Data are shown as mean ± standard derivation, *N* = 10

For AEE, ACE and AFE, high fat diet group and simvastatin group had similar responses, while 4.5 weeks treatment of AEE, ACE or AFE did not seem to have as significant hypolipidemic effect as AAE: the only parameter seems to be significantly affected was HDL, in which AEE 2 g/kg, AFE 150 and 300 mg/kg significantly increased HDL concentration relative to high fat diet group. When the treatment lasted for ten weeks, both doses (1 and 2 g/kg) AEE was found effective increasing HDL content, while only 2 g/kg AEE remarkably decreased TC, TG and LDL levels. ACE did not seem to possess hypolipidemic effects since no significant difference between ACE groups (100 and 200 mg/kg) and high fat diet group was observed. AFE, on the other hand, was found to be the most effective one among the three, effectively decreased TC, TG and LDL levels, and increased HDL levels. The effects on TG and HDL levels were dose-dependent (Tables 1, 2, 3 and 4).

### Antioxidant effect of AREs

To evaluate the direct effect of AREs on high fat diet induced oxidative stress, the levels of NO, MDA, SOD, GSH, NADPH and GSH-Px in serums of treated quails were assessed, and the results were reported in Tables 5, 6, 7 and 8. High fat diet significantly increased MDA levels and decreased NO, SOD, GSH, NADPH and GSH-Px levels relative to control group. Simvastatin effectively reverted these changes. For AAE, lowest dose (0.75 g/kg) did not significantly affect NO or SOD levels, but remarkably decreased MDA levels and increased GSH, NADPH and GSH-Px levels. Higher doses (1.5 and 3 g/kg) significantly decreased MDA levels while increased NO, GSH, NADPH and GSH-Px levels. For AEE, ACE and AFE, all the three at all doses tested significantly decreased MDA levels while increased NO, SOD, GSH, NADPH and GSH-Px levels.

**Table 5:** Nitric oxide content and antioxidant status in high fat diet-fed quails treated with AAE for ten weeks (Part I)

Treatment (/kg/day)	NO(μmol/ml)	MDA(nmol/ml)	SOD(U/ml)
Control	35.10 ± 7.79	9.21 ± 2.16	215.72 ± 60.74
Model	29.92 ± 6.94*	11.92 ± 3.13*	194.15 ± 62.45*
Simvastatin 15 mg	37.50 ± 4.79^#^	6.50 ± 1.33^#^	238.05 ± 53.76^#^
AAE 0.75 g	32.71 ± 11.27	9.29 ± 3.74^#^	205.82 ± 67.44
AAE 1.5 g	38.53 ± 9.55^#^	8.89 ± 3.66^#^	222.46 ± 70.11^#^
AAE 3 g	39.14 ± 9.35^#^	7.51 ± 2.23^#^	234.73 ± 61.82^#^

^*^statistically different from control group (*P* < 0.05)

^#^statistically different from model group (*P* < 0.05)

Male quails (three weeks old at the beginning of experiments, four weeks old at the beginning of treatments) were subjected to high fat diet (1 % cholesterol and 14 % pork oil) for a total of ten weeks. After ten weeks, quails were sacrificed and blood were collected. Nitric oxide (NO), malondialdehyde (MDA), superoxide dismutase (SOD), catalase (CAT), glutathione (GSH), nicotinamide adenine dinucleotide phosphate (NADPH) and glutathione peroxidase (GSH-Px) were measured with commercial kits following manufacturer’s protocols. Data are expressed as mean ± standard derivation, *N* = 10

**Table 6:** Nitric oxide content and antioxidant status in high fat diet-fed quails treated with AAE for ten weeks (Part II)

Treatment (/kg/day)	CAT(U/ml)	GSH(mg/L)	NADPH(μmol/ml)	GSH-Px(U)
Control	2.05 ± 0.69	3.24 ± 0.76	3.05 ± 0.95	680.65 ± 185.44
Model	1.62 ± 0.64*	2.92 ± 0.83*	2.60 ± 0.85	594.67 ± 169.58*
Simvastatin 15 mg	2.21 ± 0.73^#^	3.50 ± 1.05^#^	3.65 ± 1.10^#^	826.15 ± 185.68^#^
AAE 0.75 g	2.11 ± 0.57^#^	3.27 ± 0.74^#^	5.10 ± 1.05^#^	785.48 ± 207.43^#^
AAE 1.5 g	2.03 ± 0.65^#^	3.39 ± 0.77^#^	4.70 ± 1.35^#^	828.43 ± 190.19^#^
AAE 3 g	2.17 ± 0.85^#^	3.51 ± 0.83^#^	3.75 ± 1.20^#^	814.72 ± 169.22^#^

^*^statistically different from control group (*P* < 0.05)

^#^statistically different from model group (*P* < 0.05)

Male quails (three weeks old at the beginning of experiments, four weeks old at the beginning of treatments) were subjected to high fat diet (1 % cholesterol and 14 % pork oil) for a total of ten weeks. After ten weeks, quails were sacrificed and blood were collected. Nitric oxide (NO), malondialdehyde (MDA), superoxide dismutase (SOD), catalase (CAT), glutathione (GSH), nicotinamide adenine dinucleotide phosphate (NADPH) and glutathione peroxidase (GSH-Px) were measured with commercial kits following manufacturer’s protocols. Data are expressed as mean ± standard derivation, *N* = 10

**Table 7:** Nitric oxide content and antioxidant status in high fat diet-fed quails treated with AEE, ACE or AFE for ten weeks (Part I)

Treatment (/kg/kday)	NO(μmol/ml)	MDA(nmol/ml)	SOD(U/ml)
Control	34.89 ± 7.09	9.33 ± 1.43	210.7 ± 25.4
Model	28.54 ± 6.64*	12.13 ± 2.22*	187.1 ± 23.4*
Simvastatin 15 mg	35.79 ± 5.97^#^	7.87 ± 1.54^#^	235.5 ± 16.8^#^
AEE 1 g	34.88 ± 7.12^#^	9.40 ± 1.31^#^	215.5 ± 17.7*
AEE 2 g	35.38 ± 5.60^#^	9.30 ± 1.40^#^	217.2 ± 18.9*
ACE 100 mg	35.12 ± 5.60^#^	9.37 ± 1.19^#^	215.3 ± 20.9*
ACE 200 mg	35.84 ± 6.70^#^	9.27 ± 1.18^#^	216.3 ± 20.8*
AFE 150 mg	35.58 ± 7.91^#^	9.27 ± 1.20^#^	215.8 ± 17.8*
AFE 300 mg	36.51 ± 7.65^#^	9.11 ± 1.55^#^	220.2 ± 22.3*

^*^statistically different from control group (*P* < 0.05)

^#^statistically different from model group (*P* < 0.05)

Male quails (three weeks old at the beginning of experiments, four weeks old at the beginning of treatments) were subjected to high fat diet (1 % cholesterol and 14 % pork oil) for a total of ten weeks. After ten weeks, quails were sacrificed and blood were collected. Nitric oxide (NO), malondialdehyde (MDA), superoxide dismutase (SOD), catalase (CAT), glutathione (GSH), nicotinamide adenine dinucleotide phosphate (NADPH) and glutathione peroxidase (GSH-Px) were measured with commercial kits following manufacturer’s protocols. Data are expressed as mean ± standard derivation, *N* = 8

**Table 8:** Nitric oxide content and antioxidant status in high fat diet-fed quails treated with AEE, ACE or AFE for ten weeks (Part II)

Treatment (/kg/day)	CAT(U/ml)	GSH(mg/L)	NADPH(μmol/ml)	GSH-Px(U)
Control	2.05 ± 0.41	3.34 ± 0.78	3.25 ± 0.87	705.5 ± 90. 7
Model	1.68 ± 0.38*	2.98 ± 0.67*	2.68 ± 0.74*	617.3 ± 84.9*
Simvastatin 15 mg	2.17 ± 0.26^#^	3.53 ± 0.82^#^	3.64 ± 0.89^#^	757.4 ± 93.6^#^
AEE 1 g	2.18 ± 0.31^#^	3.32 ± 0.79^#^	3.34 ± 0.93^#^	710.2 ± 96.8^#^
AEE 2 g	2.22 ± 0.36^#^	3.36 ± 0.91^#^	3.41 ± 0.86^#^	718.5 ± 109.4^#^
ACE 100 mg	2.19 ± 0.30^#^	3.44 ± 0.93^#^	3.38 ± 0.85^#^	738.8 ± 90.4^#^
ACE 200 mg	2.20 ± 0.43^#^	3.48 ± 0.81^#^	3.54 ± 0.91^#^	751.1 ± 89.6^#^
AFE 150 mg	2.20 ± 0.28^#^	3.54 ± 0.85^#^	3.60 ± 0.78^#^	754.7 ± 78.4^#^
AFE 300 mg	2.23 ± 0.33^#^	3.55 ± 0.80^#^	3.63 ± 0.88^#^	763.9 ± 87.3^#^

^*^statistically different from control group (*P* < 0.05)

^#^statistically different from model group (*P* < 0.05)

Male quails (three weeks old at the beginning of experiments, four weeks old at the beginning of treatments) were subjected to high fat diet (1 % cholesterol and 14 % pork oil) for a total of ten weeks. After ten weeks, quails were sacrificed and blood were collected. Nitric oxide (NO), malondialdehyde (MDA), superoxide dismutase (SOD), catalase (CAT), glutathione (GSH), nicotinamide adenine dinucleotide phosphate (NADPH) and glutathione peroxidase (GSH-Px) were measured with commercial kits following manufacturer’s protocols. Data are expressed as mean ± standard derivation, *N* = 8

### Effect of treatment on terminal body weight

**Fig. 1:**
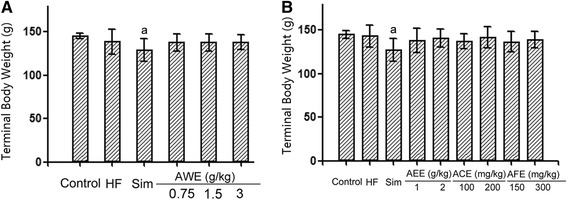
Terminal body weights of quails. Four weeks old male quails were fed with high fat diet (1 % cholesterol and 14 % pork oil, w/w) for ten weeks and then weighed for terminal body weight. **a** Body weight of quails treated with AAE (*N* = 11–17). **b** Body weight of quails treated with AEE, ACE or AFE (*N* = 11–17). a: statistically different from control group (*P* < 0.05)

No significant differences were observed among control, high diet and AREs treatment groups. The only group had a significant decrease in body weight was the simvastatin group (Fig. 1).

### Effect of AREs on aortic morphology

**Fig. 2:**
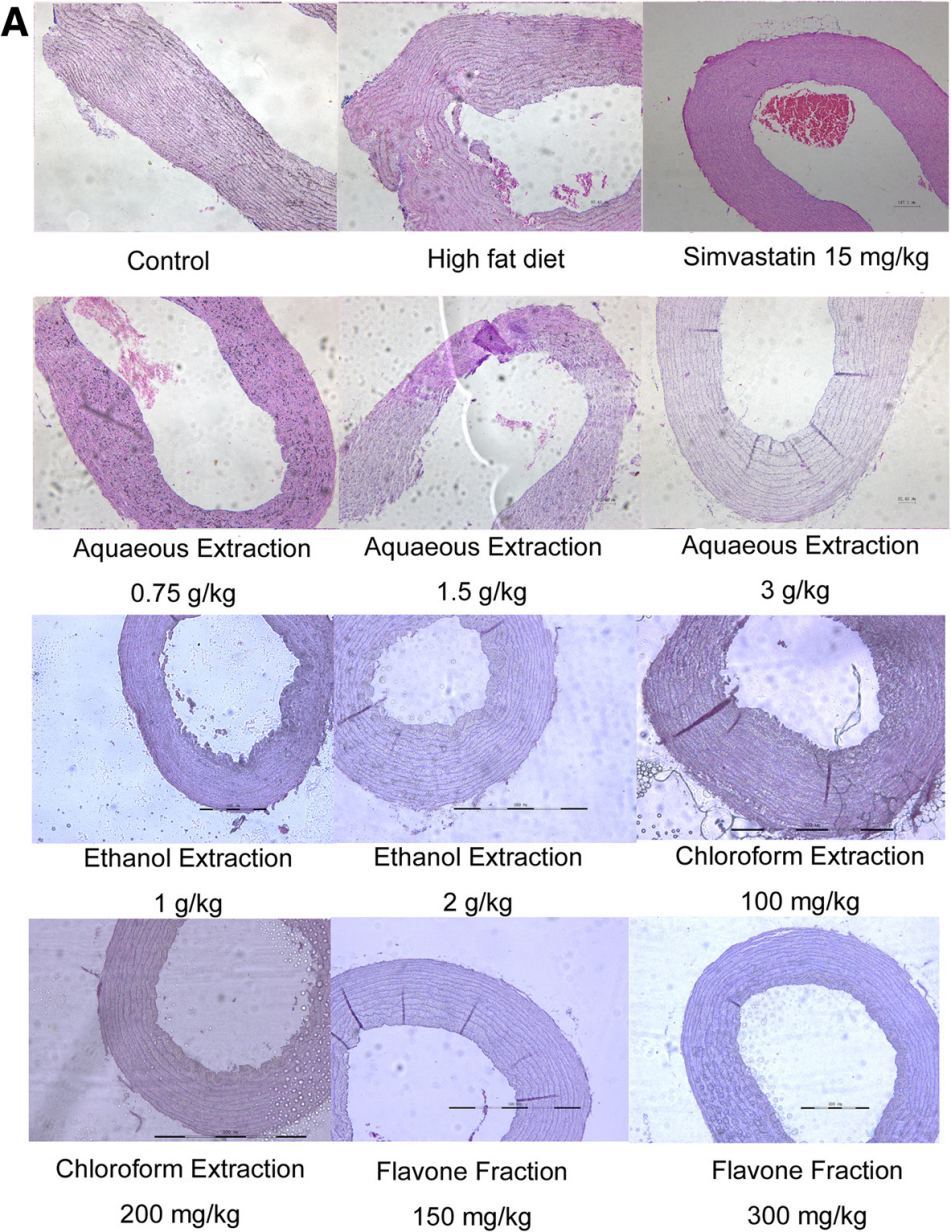
Assessment of atherosclerosis in high fat diet fed quails and the effects of AREs. Four weeks old male quails were fed with high fat diet (1 % cholesterol and 14 % pork oil, w/w) for ten weeks and then aorta were collected, fixed in 4 % formaldehyde for 24 h, then embedded in paraffin, sectioned transversally on a rotary microtome (Leica RM2016) at 6 μm and stained with hematoxlin and eosin following manufacturer’s protocol. **a** Representative pictures of aorta sections stained with hematoxylin and eosin

**Fig. 3:**
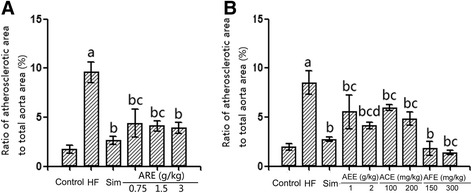
Quantification of atherosclerosis in high fat diet fed quails and the effects of AREs. Four weeks old male quails were fed with high fat diet (1 % cholesterol and 14 % pork oil, w/w) for ten weeks and then aorta were collected, fixed in 4 % formaldehyde for 24 h, then embedded in paraffin, sectioned transversally on a rotary microtome (Leica RM2016) at 6 μm and stained with hematoxlin and eosin following manufacturer’s protocol. HF: high fat diet group; Sim: simvastatin group. a: statistically different from control group. b: statistically different from high fat diet group. c: statistically different from simvastatin group. **a** Quantification of the ratio of the endothelial proliferation area to total aorta area for the AAE treated animals (*N* = 3). **b** Quantification of the ratio of the endothelial proliferation area to total aorta area for the AEE, ACE and AFE treated animals (*N* = 3). d: statistically different from AEE 1 g/kg group

The histology studies displayed that quails fed the normal diet had no significant endometrial lesions, the inner surface of the aorta was morphologically smooth, and thin, and the endothelial cells were arranged orderly (Fig. 2). While quails in the high fat diet groups showed atherosclerotic leisions, including markedly thickened and rough endothelium and irregular, proliferated smooth muscle layers. Both simvastatin and AREs significantly improved the mophology of high diet fed quail aortas. In quantification, the ratio of atherosclerotic area to total aorta area was significantly decreased by AREs and simvastatin compared with the high fat diet group (Fig. 3). Among different AREs, high dose of AAE (3 g/kg) and low dose of AFE (150 mg/kg) exhibited comparable protective effects as simvastatin; high dose of AFE (300 mg/kg) treatment resulted in an even greater anti-atherosclerosis effect comparing to simvastatin.

### Effect of AREs on aortic ultrastructure

**Fig. 4:**
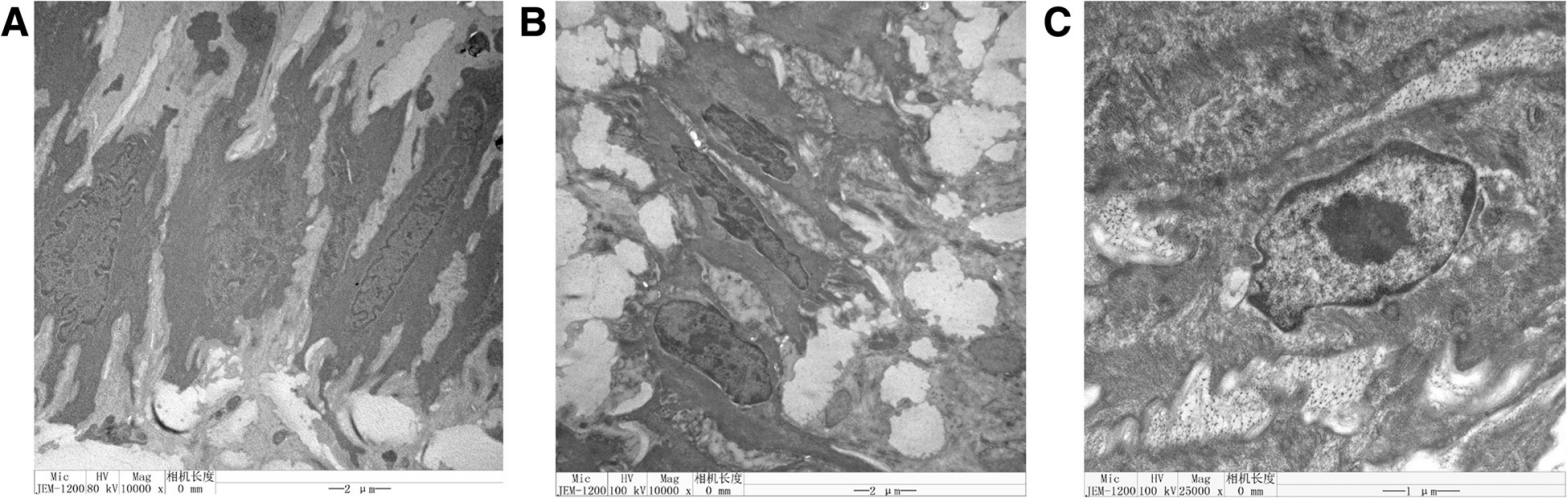
Ultrastructure of quail aorta in high fat diet fed quails and the effects of AREs. Four weeks old male quails were fed with high fat diet (1 % cholesterol and 14 % pork oil, w/w) for ten weeks and then aorta were collected, fixed in 5 % glutaraldehyde for 24 h, dehydrated with graded ethanol, embedded with epoxy resin 618 and then sectioned and observed under a transmission electronic microscope JEM-1200EX. **a** representative TEM picture of control quail aorta. **b** representative TEM picture of high fat diet fed quail aorta. **c** representative TEM picture of AFE 300 mg/kg/d treated quail aorta

Similar to previously reported ultrastructure changes in atherosclerosis [21], high fat diet resulted in deterioration of ultrastructure of quail aorta under TEM, including vacuolation and diminished ultrastructure. Treatment with AREs effectively improved the ultrastructure, among which AFE seemed to have the best protective effects (Fig. 4).

### Effect of AREs on aortic lipids

**Fig. 5:**
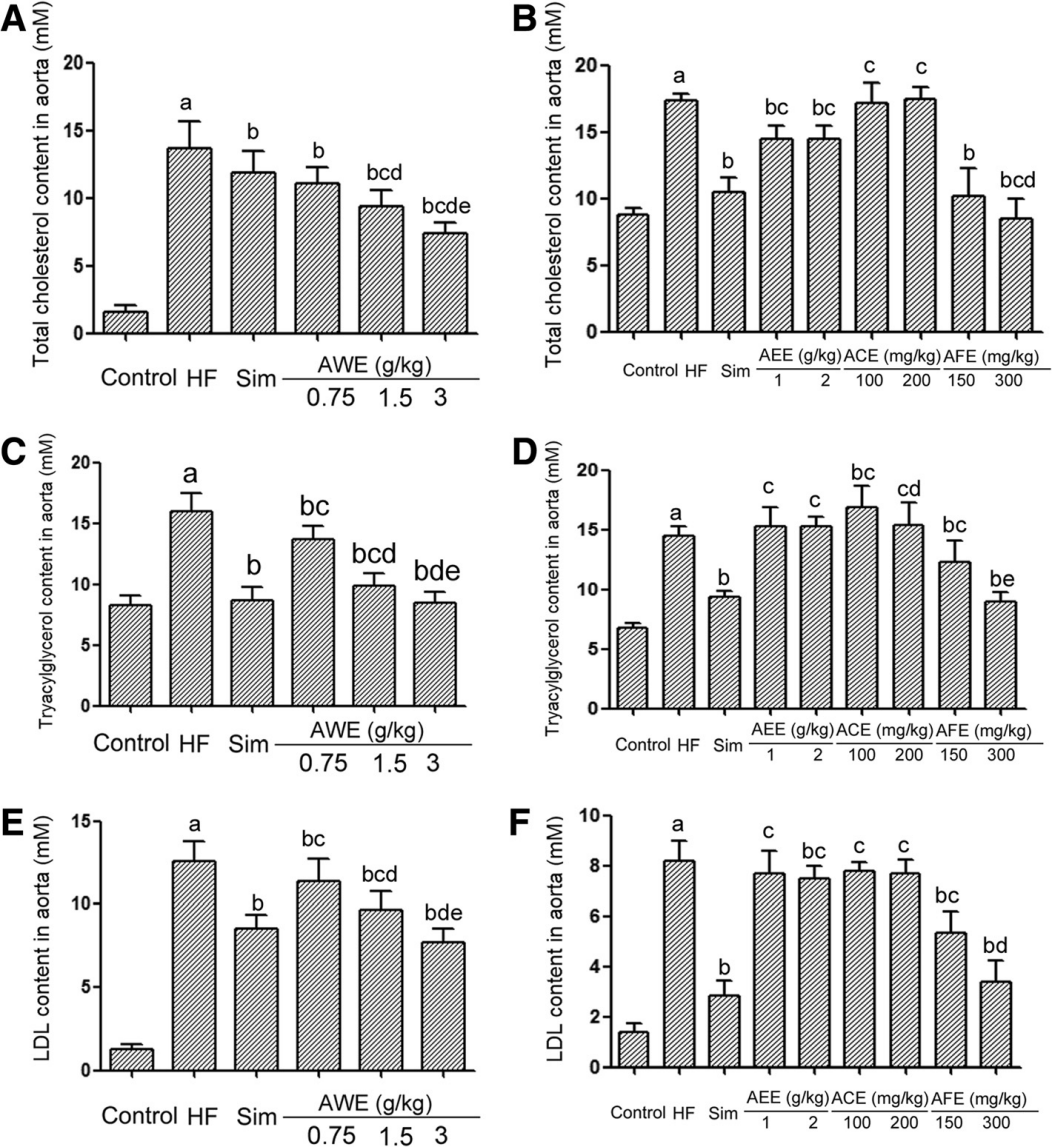
Lipid profile of aorta in high fat diet fed quails and the effects of AREs. Four weeks old male quails were fed with high fat diet (1 % cholesterol and 14 % pork oil, w/w) for ten weeks and then aorta were collected, lipids were extracted from homogenized aorta tissue with 1,4-dioxane and the lipid contents were assessed with an automatic biochemical analyzer (Beckman AU5400, Brea, CA, US) . HF: high fat diet group; Sim: simvastatin group. a: statistically different from control group. b: statistically different from high fat diet group. c: statistically different from simvastatin group. **a** Total cholesterol content in aorta for AAE treated animals (*N* = 10). d: statistically different from AAE 0.75 g/kg group. e: statistically different from AAE 1.5 g/kg group. **b** Total cholesterol content in aorta for AEE, ACE and AFE treated animals (*N* = 10). d: statistically different from AFE 150 mg/kg group. **c** Triacylglycerol content in aorta for AAE treated animals (*N* = 10). d: statistically different from AAE 0.75 g/kg group. e: statistically different from AAE 1.5 g/kg group. **d** Triacylglycerol content in aorta for AEE, ACE and AFE treated animals (*N* = 10). d: statistically different from ACE 100 mg/kg group. e: statistically different from AFE 150 mg/kg group. **e** LDL content in aorta for AAE treated animals (*N* = 10). d: statistically different from AWE 0.75 g/kg group. e: statistically different from AWE 1.5 g/kg group. **f** LDL content in aorta for AEE, ACE and AFE treated animals (*N* = 10). d: statistically different from AFE 150 mg/kg group

Lipid profile including TC, TG and LDL were assessed in aorta samples. As shown in Fig. 5 left columns, the levels of TC, TG and LDL were significantly decreased by simvastatin as well as all doses of AAE (0.75, 1.5 and 3 g/kg). The hypolipidemic effect of AAE is dose-dependent. On the other hand, as demonstrated in Fig. 5 right columns, ACE did not seem to have hypolipidemic effect, actually, it seemed to have limited hyperlipidemic effect, increased TG level slightly. AEE had no significant effect on TG level, but reduced TC level effectively, though to a much lesser extent comparing to simvastatin. High dose of AEE (2 g/kg) could also decrease LDL level. AFE effectively decreased the levels of TC, TG and LDL, with a similar pattern as AAE.

## Discussion

### Hypolipidemic effects of AREs

Hyperlipidemia, especially increased TC, TG and LDL levels, along with decreased HDL level, are major risk factors for atherosclerosis [22]. Many current drugs, such as statins, mainly target the blood lipids to prevent/treat atherosclerosis. Among the four AREs tested in the current study, ACE does not possess significant hypolipidemic effect and AEE only possess limited effect, while AAE and AFE possess significant hypolipidemic effects, whose onset seems to be a bit slower comparing to simvastatin, but with enough administration duration, the hypolipidemic effects are comparable to the well-characterized hypolipidemic agent simvastatin, thus AAE or AFE administration has the potential to decrease the risk of atherosclerosis.

### NO and antioxidant status

NO plays a key role in the pathogenesis of vascular diseases [23, 24]. It is well documented that endothelial dysfunction caused by lack of endothelium-derived NO production and/or decreased nitric oxide (NO) bioavailability or activity has been linked to atherosclerosis initiation and progression [25, 26] Moreover, NO deficiency can directly impair vascular function and structure thus promote arteriosclerosis [27]. The results from current study demonstrated that, similar with simvastatin, AREs (except for 0.75 g/kg AAE) increases NO production significantly. Thus, the increase of NO production could also contribute to the anti-atherosclerosis effect of AAE.

It has been demonstrated that oxidative stress plays a key role in the initiation and progression of atherosclerosis. The generation of oxidative stress may induce vascular disorder and contribute to atherosclerotic plaque formation [28]. One of the outcome of oxidative stress, the lipid peroxidation, could damage the cell plasma membrane and further leads to deletion of cytoplasmic components and cell death [28]. MDA, an end product of lipid peroxidation, is considered a critical biomarker of oxidative stress [29]. Enzymes including SOD, CAT and GPx, can reduce the ROS level [30–32]. In current study, the levels of MDA were significantly increased after high fat diet, confirming high levels of oxidative stress. Meanwhile, the levels of anti-oxidant related enzymes SOD, CAT and GSH-Px, as well as endogenous reducing agents GSH and NADPH were significantly decreased, further confirming the induction of oxdative stress by high fat diet. AREs at all doses (except for the lowest dose on SOD levels) significantly reverted the changes, decreasing the levels of MDA and increasing the levels of SOD, CAT, GSH-Px, GSH and NADPH. Therefore, endogenous antioxidants regulation is a possible mechanism involved in the anti-atherosclerosis effects of AREs.

### Atherosclerotic changes

Consistent with published results with atrovastatin [33], both simvastatin and AREs significantly improved the mophology of high diet fed quail aortas. This is the direct confirmation that AREs could protect high fat diet fed quail against atherosclerosis formation in aorta. Among different AREs, high dose of AAE (3 g/kg) and low dose of AFE (150 mg/kg) exhibited comparable protective effects as simvastatin; high dose of AFE (300 mg/kg) treatment resulted in an even greater anti-atherosclerosis effect comparing to simvastatin. The protective effects were further confirmed with TEM results, in which AFE exerted protective effects for the microstructures of aorta. These data indicates great potential for the AFE fraction to be used as anti-atherosclerosis agents.

### Aorta lipid profile

The lipid contents of aorta directly reflects the amount of lipid deposition. Technically, the lower aorta lipid profile is, the less lipid has deposited in the aorta. Our results indicates that AAE and AFE exerted best hypolipidemic effects among the four AREs tested, which is consistent with our morphological assessment. Interestingly, AAE seems to be more potent decreasing TC, with 1.5 and 3 g/kg decreased TC even further than simvastatin did. Meanwhile, high dose AAE (3 g/kg) are about as potent as simvastatin reducing TG and LDL. The differential response in TC and TG/LDL suggests that AAE are more effective reducing cholesterol other than LDL. Further mechanistic study is planned to explore this effect. Another point worth noting is that even ACE, which did not have any hypolipidemic effect, exhibited some antiatherosclerosis effect, suggesting the existence of hypolipidemic effect-independent mechanism, which might worth further exploration.

## Conclusions

To evaluate the protective effects of AAE on high fat diet induced atherosclerosis, the high fat diet induced quail atherosclerosis model was successfully established and used for the investigation. The results revealed that AAE is effective in protection against high fat diet induced weight gain, improving serum lipid profile (decreasing serum LDL, TG and TC levels, increasing serum HDL level); protecting against oxidative stress (decreasing MDA level, while increasing SOD, GSH, GSH-Px and CAT levels), increasing NO levels in serum, decreasing lipid content in aorta, and decreasing atherosclerotic area in the aorta. All these effects indicate that AAE is a promising agent in the prevention of atherosclerosis. The underlying molecular mechanism is currently under investigation.
